# Comparative analysis of laparoscopic internal ring reinforcement combined with high ligation versus double-loop high ligation in the treatment of pediatric giant inguinal hernia

**DOI:** 10.1186/s12893-026-04056-y

**Published:** 2026-07-21

**Authors:** Qingbao He, Hongjia He, Kaisheng Li, Xiaoyang Liu

**Affiliations:** Department of Minimally Invasive Urologic Surgery, Children’s Hospital Affiliated to Shandong University, Jinan, Shandong China

**Keywords:** Laparoscopy, Pediatric inguinal hernia, Giant inguinal hernia, Internal ring reinforcement, High ligation

## Abstract

**Objective:**

To evaluate the clinical outcomes of laparoscopic internal ring reinforcement combined with high ligation and double-loop high ligation in the treatment of pediatric giant inguinal hernia.

**Methods:**

This retrospective cohort study included 381 children with giant inguinal hernia who underwent laparoscopic repair at the Children’s Hospital Affiliated to Shandong University between February 2021 and April 2025. Patients were categorized into three groups according to the surgical technique employed. Group A (*n* = 155) underwent laparoscopic internal ring reinforcement by suturing the iliopubic tract to the lower margin of the transversus abdominis arch combined with high ligation of the hernia sac. Group B (*n* = 130) underwent laparoscopic double-loop high ligation, whereas the control group (*n* = 96) underwent conventional single high ligation. Perioperative outcomes, including operative time, intraoperative subperitoneal hematoma, 24-hour postoperative pain score, and recurrence within 12 months, were compared among the groups. Given the retrospective nature of the study and the potential for treatment-selection bias, an exploratory subgroup analysis was additionally performed in patients with an internal ring diameter of 1.5–2.0 cm.

**Results:**

No significant differences were observed among the three groups with respect to age, sex, laterality, or history of incarceration (all *P* > 0.05). Using a 2.0-cm threshold derived from institutional experience, patients with an internal ring diameter ≥ 2.0 cm preferentially underwent reinforcement-based procedures. Operative time was significantly longer in Groups A and B than in the control group (*P* < 0.001). During the 12-month follow-up period, no recurrence was observed in either reinforcement group, whereas three recurrences occurred in the control group, resulting in a significant difference among the groups (*P* = 0.015). No significant differences were identified in the incidence of intraoperative subperitoneal hematoma or 24-hour postoperative pain scores (*P* = 0.946 and *P* = 0.589, respectively). In the exploratory subgroup analysis of patients with an internal ring diameter of 1.5–2.0 cm, recurrence remained less frequent in the reinforcement groups, while postoperative pain scores and hematoma rates were comparable among the three groups. Exploratory odds ratio analysis also suggested a potential association between reinforcement-based procedures and a lower observed recurrence rate.

**Conclusions:**

In children with giant inguinal hernia, reinforcement-based laparoscopic procedures may be associated with a lower observed recurrence rate than conventional single high ligation, without an apparent increase in operative morbidity. Double-loop high ligation may represent a feasible reinforcement option when excessive tension is encountered after single high ligation. These findings suggest a potential benefit of an internal ring diameter-oriented surgical strategy in selected patients. However, the 2.0-cm threshold used in this study was based on institutional experience rather than formal cutoff validation, and the results should be interpreted cautiously given the retrospective design, potential treatment-selection bias, and the limited number of recurrence events. Further prospective multicenter studies are required to validate these findings.

## Introduction

Pediatric indirect inguinal hernia is one of the most common conditions encountered in pediatric surgery and results from incomplete obliteration of the processus vaginalis during embryonic development. Following birth, increases in intra-abdominal pressure caused by crying, constipation, or physical activity may facilitate protrusion of abdominal contents, such as bowel loops or omentum, through the internal inguinal ring into the inguinal canal or scrotum [1]. Based on previous studies, pediatric giant inguinal hernia is generally defined as an internal ring diameter greater than 1.5 cm measured intraoperatively [2, 3]. For conventional (non-giant) inguinal hernias, laparoscopic high ligation (LHL) has become a widely accepted treatment owing to its favorable clinical outcomes and low recurrence rate [4]. However, in patients with giant inguinal hernia, the relatively large internal ring defect and increased tissue tension after ligation may compromise closure stability and may be associated with a higher risk of postoperative recurrence [5]. In our clinical practice, we observed that when the internal ring diameter approached or exceeded 2.0 cm, tension at the ligation site frequently increased after conventional high ligation, potentially affecting the security of closure [6]. Consequently, reinforcement-based procedures were increasingly adopted in selected patients with larger defects. Nevertheless, the optimal surgical strategy for pediatric giant inguinal hernia remains uncertain, and no consensus has been established regarding the management of large internal ring defects [7]. Furthermore, the clinical value of using the internal ring diameter to guide surgical decision-making has not been adequately investigated. Currently, two reinforcement-based laparoscopic techniques are primarily used in our institution for the treatment of giant inguinal hernia: (1) laparoscopic internal ring reinforcement by suturing the iliopubic tract to the lower margin of the transversus abdominis arch combined with high ligation of the hernia sac, and (2) laparoscopic double-loop high ligation. Both techniques are intended to reduce tension at the internal ring and enhance closure stability while maintaining acceptable procedural safety and feasibility. Therefore, this retrospective study compared these two reinforcement-based procedures with conventional laparoscopic high ligation to evaluate their potential association with postoperative outcomes in children with giant inguinal hernia. In addition, we explored whether an internal ring diameter-oriented surgical strategy may provide practical guidance for procedure selection in this patient population.

## Materials and methods

### Study population

This retrospective cohort study was conducted through the review of previously collected clinical records of children who underwent laparoscopic repair at the Department of Minimally Invasive Urologic Surgery, Children’s Hospital Affiliated to Shandong University, between February 2021 and April 2025. A total of 381 children with giant unilateral indirect inguinal hernia were included. No patients presented with bilateral giant inguinal hernia. Patients were categorized into three groups according to the surgical procedure performed. Group A underwent laparoscopic internal ring reinforcement by suturing the iliopubic tract to the lower margin of the transversus abdominis arch combined with high ligation of the hernia sac. Group B underwent laparoscopic double-loop high ligation, whereas the control group underwent conventional single laparoscopic high ligation. Because of the retrospective study design, procedure selection was based on intraoperative assessment and surgeon judgment rather than random allocation. All operations were performed by senior attending surgeons or surgeons of higher academic rank with substantial experience in pediatric laparoscopic hernia repair. This retrospective study was approved by the Institutional Review Board of the Children’s Hospital Affiliated to Shandong University on January 15, 2026 (Approval No. SDFE-IRB/T-2026008). The approval covered the retrospective review and analysis of previously collected clinical data. Because of the retrospective nature of the study, the requirement for written informed consent was waived by the Institutional Review Board.

### Inclusion and exclusion criteria

(1) Inclusion criteria: Patients were eligible for inclusion if they met all of the following criteria: (1) diagnosis of indirect inguinal hernia confirmed by ultrasonography; and (2) an internal ring diameter > 1.5 cm determined by preoperative ultrasonography and/or confirmed by intraoperative measurement. Exclusion criteria: Patients were excluded if they met any of the following criteria: (1) recurrent giant inguinal hernia or the need for intraoperative modification of the planned procedure because of severe adhesions around the internal ring; (2) intraoperative confirmation of direct inguinal hernia or the presence of concomitant conditions requiring additional surgical intervention; (3) significant respiratory, immunologic, cardiovascular, or other systemic diseases that could potentially affect perioperative outcomes; or (4) loss to follow-up during the postoperative observation period.

### Measurement of internal ring diameter

**Fig. 1:**
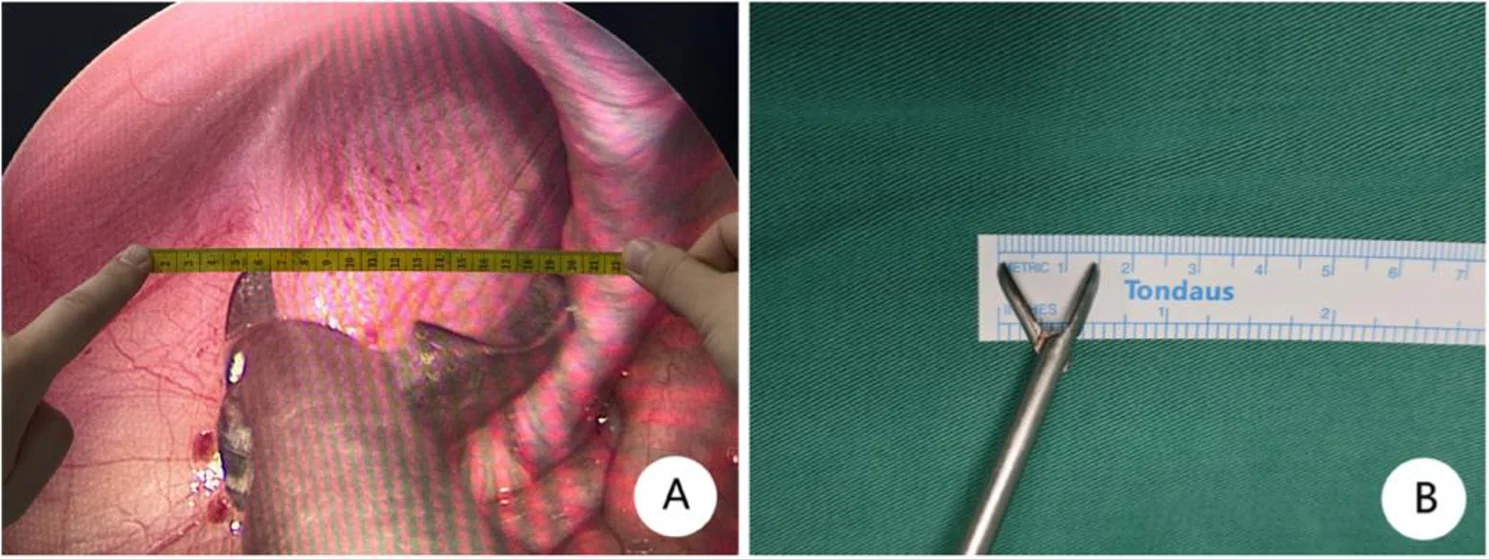
Steps of the proportional indirect measurement method. **A**: Measure the diameter of the internal inguinal ring and the opening length of the laparoscopic needle holder on the screen; **B**: Measure the actual opening length of the laparoscopic needle holder

The internal ring diameter was determined using a proportional indirect measurement method. Briefly, the jaws of a laparoscopic needle holder were fully opened and aligned with the maximal transverse diameter of the internal ring defect under laparoscopic visualization. An assistant then used a flexible ruler to measure both the apparent maximal internal ring diameter and the apparent width of the opened needle holder displayed on the monitor screen. The actual maximal opening width of the needle holder was measured separately outside the body and used as a reference value (Fig. 1). The actual internal ring diameter was subsequently calculated using the following proportional formula: Actual internal ring diameter = (Measured internal ring diameter on the monitor screen × Actual needle holder opening width) / Measured needle holder opening width on the monitor screen (Fig. 1). To improve measurement consistency, all measurements were performed intraoperatively by experienced surgeons using the same laparoscopic instruments and measurement protocol throughout the study period.

### Surgical techniques

All patients underwent general anesthesia with combined intravenous and inhalational agents and endotracheal intubation. Patients were positioned supine in a slight Trendelenburg position. Two 5-mm trocars were inserted through bilateral periumbilical incisions, and pneumoperitoneum was established using a standard laparoscopic technique.

#### Group A: internal ring reinforcement combined with high ligation (Fig. 2)

**Fig. 2:**
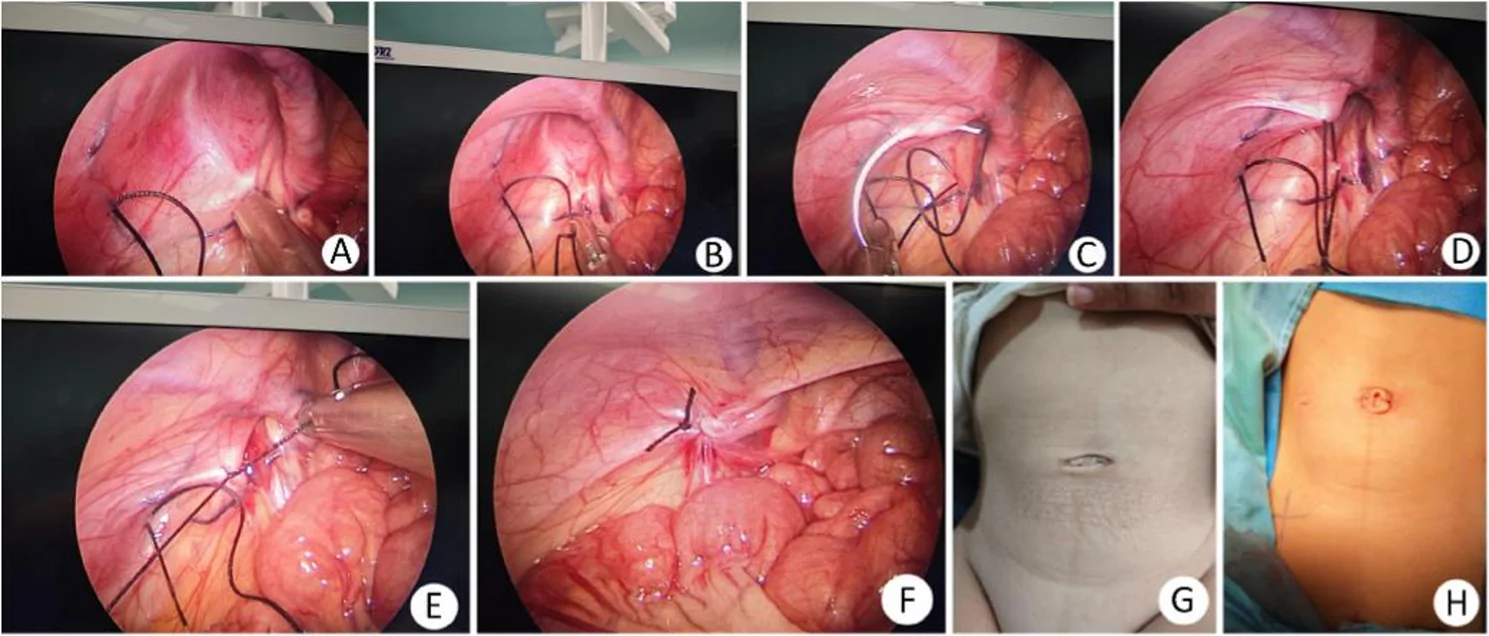
Iliopubic tract-transversus abdominis arch reinforcement. **A**: Circular suturing of the internal inguinal ring; the needle holder indicates the location of the iliopubic tract. **B**: The needle tip is pressed downward horizontally to penetrate the iliopubic tract; **C**: The needle tip is directed outward to suture the lower border of the transversus abdominis aponeurotic arch; **D**: Traction on the suture confirms successful incorporation of the ligament and muscle; **E**: Knot tying of the iliopubic tract and the transversus abdominis aponeurotic arch suture; **F**: Tightening and knot tying of the sutures around the internal inguinal ring; **G**: Concealed incision at both umbilical wheel edges; **H**: An additional 3-mm trocar incision created

Under laparoscopic visualization, the iliopubic tract was identified as a distinct whitish fibrous band located inferior to the internal ring and extending along the transversalis fascia. After careful dissection and confirmation of the anatomical landmarks, the iliopubic tract was approximated to the lower margin of the transversus abdominis arch to reduce the size of the internal ring and reinforce the posterior wall of the inguinal region. For right-sided defects, the needle was introduced from the inferolateral aspect of the iliopubic tract and exited at its superomedial aspect; the direction was reversed for left-sided defects. Intracorporeal suturing and knot tying were then performed to achieve reinforcement of the internal ring. Subsequently, a 2 − 0 silk suture mounted on a round-bodied needle was introduced percutaneously through an avascular area superolateral to the internal ring. A circumferential purse-string suture was placed around the internal ring while carefully avoiding injury to the vas deferens and spermatic vessels. Extracorporeal knot tying was then performed to complete high ligation of the hernia sac. In cases with prominent or deeply recessed peritoneal folds adjacent to the spermatic cord structures, temporary suspension sutures were placed through either the ipsilateral or contralateral abdominal wall to elevate and flatten the folds. This maneuver facilitated complete circumferential closure of the peritoneum while minimizing the risk of inadvertent entrapment of the vas deferens or spermatic vessels within the ligature.

#### Group B: double-loop high ligation (Fig. 3)

**Fig. 3:**
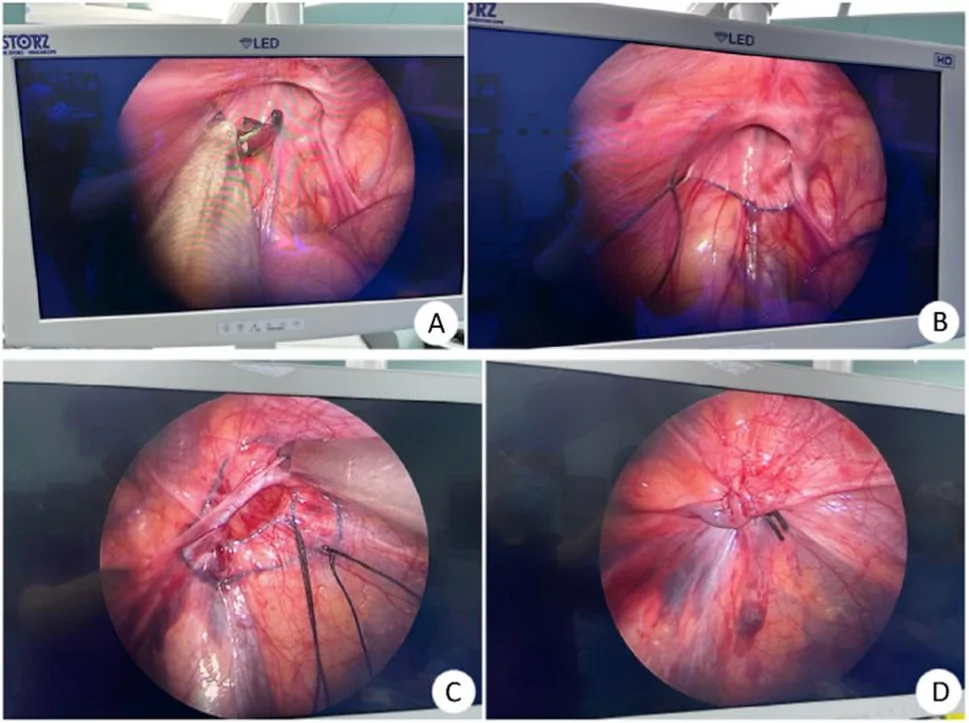
Laparoscopic double-loop high ligation of the hernia sac. **A**: Measurement of the internal inguinal ring diameter using the proportional indirect measurement method; **B**: A 2 − 0 silk suture was placed circumferentially around the internal inguinal ring while carefully avoiding the spermatic vessels and vas deferens, followed by moderate tightening of the first ligature; **C**: A second purse-string suture was placed proximally to the first ligature, ensuring that the two ligatures remained independent and non-intersecting; **D**: After placement of the first ligature around the internal inguinal ring, a second independent ligature was placed proximally, and the two sutures were tied separately to reinforce closure

Patient positioning, trocar placement, and pneumoperitoneum establishment were identical to those used in Group A. A first circumferential purse-string suture using 2 − 0 silk was placed around the internal ring while preserving the vas deferens and spermatic vessels. After moderate tightening of the first ligature, a second independent purse-string suture was placed proximal to the first. Care was taken to ensure that the two ligatures remained separate and non-overlapping before individual extracorporeal knot tying was performed.

#### Control group: conventional single high ligation (Fig. 4)

**Fig. 4:**
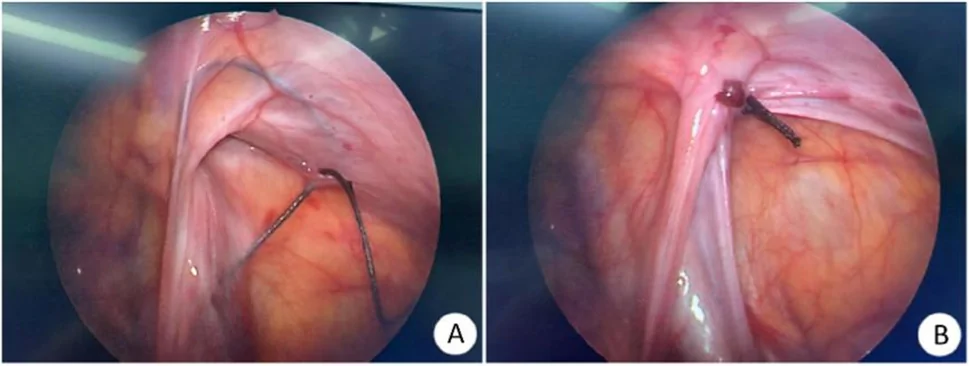
Laparoscopic high ligation of the hernia sac. **A**: A 2 − 0 silk suture with a round-bodied needle was introduced percutaneously through an avascular area superolateral to the internal ring, and circumferential high ligation of the internal ring peritoneum was performed under laparoscopic guidance; **B**: Care was taken to avoid omission of peritoneal folds and to prevent incorporation of the vas deferens, spermatic vessels, or surrounding muscle tissue into the ligature

A 2 − 0 silk suture mounted on a round-bodied needle was introduced through an avascular area superolateral to the internal ring. Under laparoscopic guidance, the peritoneum surrounding the internal ring was circumferentially encircled and ligated while carefully preserving the vas deferens, spermatic vessels, and adjacent muscular structures. Particular attention was paid to avoiding omission of peritoneal folds during ligation. After knot tying, the internal ring was re-examined laparoscopically to confirm complete closure without any visible residual defect. Adequate closure was considered achieved when no residual opening or peritoneal gap was observed. All procedures were performed by the same surgical team.

To minimize inter-operator variability, all surgeons had extensive experience in pediatric laparoscopic inguinal hernia repair and had completed the learning curve for advanced laparoscopic hernia procedures before participating in the study.

### Outcome measures

The following variables were evaluated: age (months), sex, internal ring diameter, history of incarceration, operative time, incidence of intraoperative subperitoneal hematoma, postoperative pain score at 24 h, and recurrence within 12 months after surgery. Operative time was defined as the duration of the unilateral procedure, measured from needle insertion to completion of knot tying. Intraoperative subperitoneal hematoma was defined as localized bleeding resulting from inadvertent injury to small subperitoneal vessels during suturing that was identifiable under laparoscopic visualization. Postoperative pain at 24 h was assessed using the FLACC (Face, Legs, Activity, Cry, and Consolability) scale, a validated behavioral pain assessment tool commonly used in pediatric patients. The FLACC scale evaluates five domains, including facial expression, leg movement, activity, crying, and consolability [8]. Each domain is scored from 0 to 2, resulting in a total score ranging from 0 to 10, with higher scores indicating greater pain severity. Recurrence was defined as the reappearance of a clinically detectable inguinal or scrotal bulge on the operated side, with or without symptoms, and was confirmed by ultrasonographic examination during follow-up. All patients underwent scheduled postoperative follow-up at 1, 3, 6, and 12 months after surgery.

### Statistical analysis

Statistical analyses were performed using IBM SPSS Statistics version 27.0 (IBM Corp., Armonk, NY, USA). Continuous variables were assessed for normality using the Shapiro–Wilk test and for homogeneity of variance using Levene’s test. Variables satisfying both assumptions were expressed as mean ± standard deviation (SD) and compared using one-way analysis of variance (ANOVA), whereas variables that did not satisfy these assumptions were expressed as median (interquartile range [IQR]) and analyzed using the Kruskal–Wallis H test. Categorical variables were compared using the chi-square test or Fisher’s exact test, as appropriate. All statistical tests were two-sided, and *P* < 0.05 was considered statistically significant. To partially reduce treatment-selection bias inherent to the retrospective study design, an exploratory subgroup analysis was performed in patients with an internal ring diameter of 1.5–2.0 cm. Because postoperative recurrence events were infrequent, multivariable regression analysis and propensity-score adjustment were considered statistically unreliable and therefore were not performed. Instead, exploratory odds ratios (ORs) with 95% confidence intervals (CIs) were calculated for recurrence outcomes. A post hoc power analysis for the primary recurrence outcome was additionally performed using G*Power version 3.1 (Heinrich Heine University, Düsseldorf, Germany) with a two-sided significance level of α = 0.05. This study was prepared and reported in accordance with the Strengthening the Reporting of Observational Studies in Epidemiology (STROBE) guidelines [9].

## Results

All patients completed the scheduled postoperative follow-up visits at 1, 3, 6, and 12 months after surgery, including ultrasonographic evaluation, and no patient was lost to follow-up. The median follow-up duration was 12 months. All surgical incisions healed uneventfully without wound-related complications. No significant differences were observed among Group A, Group B, and the control group with respect to age, sex, laterality, or history of incarceration (all *P*>0.05), indicating satisfactory baseline comparability (Table 1). All continuous variables satisfied the assumptions of normality and homogeneity of variance; therefore, comparisons of age, operative time, and postoperative FLACC pain scores were performed using one-way analysis of variance (ANOVA). Regarding the internal ring diameter, patients with defects ≥ 2.0 cm preferentially underwent reinforcement-based procedures according to institutional practice because larger defects were considered more likely to be associated with increased tension after ligation and a potentially higher risk of recurrence. The mean operative time was 21.37 ± 3.55 min in Group A, 20.95 ± 4.17 min in Group B, and 15.69 ± 3.13 min in the control group. Operative time was significantly longer in both reinforcement groups than in the control group (*P*<0.001). Intraoperative subperitoneal hematoma occurred in 3 patients in Group A (maximum diameter, 1.5 cm), 2 patients in Group B (maximum diameter, 1.0 cm), and 2 patients in the control group (maximum diameter, 1.8 cm). All cases resulted from minor injury to small subperitoneal vessels during suturing, and no active bleeding was observed at the completion of surgery. No significant difference was detected among the three groups in the incidence of subperitoneal hematoma (*P*=0.946). No significant differences were observed among the three groups in postoperative 24-hour FLACC pain scores (*P*=0.589). Pain severity gradually improved before discharge and resolved completely during follow-up in all patients. During the 12-month follow-up period, no recurrence was observed in either reinforcement group, whereas three recurrences occurred in the control group. The difference in recurrence rates among the three groups reached statistical significance (*P*=0.015) (Table 2). Exploratory odds ratio analysis yielded findings consistent with the primary analysis and suggested a potential association between reinforcement-based procedures and lower observed recurrence rates (Table 3). Post hoc power analysis for the primary recurrence outcome demonstrated a statistical power of 85.4%. To partially address potential treatment-selection bias, an exploratory subgroup analysis was performed in patients with an internal ring diameter of 1.5–2.0 cm (Table 4). Within this subgroup, operative time remained significantly longer in Groups A and B than in the control group (*P* < 0.001), whereas no significant differences were observed in the incidence of subperitoneal hematoma or postoperative pain scores (all *P* > 0.05). During follow-up, no recurrence occurred in either the reinforcement group, whereas three recurrences occurred in the control group (*P*=0.022). Overall, the subgroup analysis showed findings generally consistent with those of the primary analysis. All three recurrent cases occurred within 6 months after surgery. The internal ring diameter in these patients was close to 2.0 cm, and one patient had a previous history of incarceration (Table 5). All recurrent patients subsequently underwent reoperation. Laparoscopic findings at reoperation suggested incomplete closure of the internal ring and increased local tension at the ligation site as possible contributing factors; however, definitive causality could not be established. No further recurrence was observed during subsequent follow-up.

**Table 1:** Comparison of general characteristics and intraoperative findings among children in study Group A, Study Group B, and the control group

Variable	Study Group A (*n* = 155)	Study Group B (*n* = 130)	Control Group (*n* = 96)	Statistical Value	*P* Value
Age (months), mean ± SD	25.96 ± 8.36	26.73 ± 8.69	26.55 ± 7.97	F = 0.328	0.721
Sex, n (%)				χ2 = 1.053	0.591
Female	18	12	13		
Male	137	118	83		
Side, n (%)				χ2 = 2.080	0.354
Right	30	20	22		
Left	125	110	74		
History of incarceration, n (%)				χ2 = 1.153	0.562
Yes	85	67	46		
No	70	63	50		
Internal ring diameter, n (%)				χ2 = 22.727	< 0.001
1.5–2 cm	122	109	96		
≥ 2 cm	33	21	0		

**Table 2:** Comparison of surgical conditions among Group A, Group B, and the control group

Variable	Study Group A (*n* = 155)	Study Group B (*n* = 130)	Control Group (*n* = 96)	Statistic	*P* value
Operative time (min)	21.37 ± 3.55	20.95 ± 4.17	15.69 ± 3.13	F = 80.447	< 0.001
subperitoneal hematoma				χ2 = 0.112	0.946
Yes	3	2	2		
No	152	128	94		
Pain score	2.24 ± 0.75	2.23 ± 0.67	2.15 ± 0.69	F = 0.530	0.589
Recurrence					0.015
Yes	0	0	3		
No	155	130	93		

**Table 3:** Odds ratio analysis of postoperative recurrence

Case	Recurrence rate, *n*/*N* (%)	OR	95% CI	*P* value
Reinforcement-based procedures (Groups A and B)	0/285 (0%)	0.05	0.002–0.98	0.015
Conventional high ligation	3/96 (3.1%)	Reference	-	-

**Table 4:** Subgroup analysis of perioperative outcomes among patients with an internal ring diameter of 1.5–2.0 cm

Variable	Study Group A (*n* = 122)	Study Group B (*n* = 109)	Control Group (*n* = 96)	Statistic	*P* value
Operative time (min)	20.83 ± 2.70	20.12 ± 3.86	15.69 ± 3.13	F = 75.498	< 0.001
subperitoneal hematoma				χ2 = 0.062	0.969
Yes	2	2	2		
No	120	107	94		
Pain score	2.03 ± 0.52	1.97 ± 0.60	2.15 ± 0.69	F = 2.356	0.096
Recurrence					0.022
Yes	0	0	3		
No	122	109	93		

**Table 5:** Clinical characteristics of patients with postoperative recurrence

Case	Age (months)	Internal ring diameter	History of incarceration	Surgical procedure	Time to recurrence (months)	
1	18	1.8	No	Single high ligation	3	
2	53	1.9	Yes	Single high ligation	5	
3	66	1.8	No	Single high ligation	5	

## Discussion

With the widespread adoption of minimally invasive surgery, laparoscopic repair has become the preferred treatment for pediatric indirect inguinal hernia in many institutions because of its advantages, including improved cosmetic outcomes, reduced surgical trauma, faster recovery, and the ability to simultaneously identify and manage a contralateral patent processus vaginalis [10–11]. Currently, the two principal laparoscopic approaches are intracorporeal high ligation and percutaneous extraperitoneal high ligation, both of which are based on closure of the patent or incompletely obliterated processus vaginalis [12, 13]. Previous studies have demonstrated that laparoscopic repair achieves outcomes comparable or superior to those of conventional open repair, with reported recurrence rates ranging from 0.13% to 0.86% in contemporary series [14−15]. In recent years, percutaneous internal ring suturing (PIRS) has also gained increasing popularity because of its technical simplicity and favorable clinical outcomes [16]. Several studies have identified the internal ring diameter as an important factor associated with postoperative recurrence following laparoscopic repair [17]. Han et al. [18] classified internal ring defects into three categories according to diameter: type I (> 1.5 cm), type II (0.5–1.5 cm), and type III (< 0.5 cm). Type I defects demonstrated a significantly higher recurrence rate than smaller defects, suggesting that internal ring size should be considered during surgical planning. Current evidence indicates that recurrence after pediatric laparoscopic hernia repair may be related to multiple factors, including insecure knot tying or suture loosening, omission of peritoneal folds during ligation, laxity of the abdominal wall and surrounding peritoneum, and excessive tension generated during closure of large internal ring defects [19–22].

In the present study, both reinforcement-based procedures required significantly longer operative times than conventional single high ligation (*P* < 0.001), reflecting increased technical complexity. Previous studies have demonstrated that pediatric laparoscopic inguinal hernia repair is associated with a measurable learning curve, with operative efficiency and surgical outcomes improving progressively as surgeon experience increases [23]. Pogorelić et al. [24] reported that proficiency in laparoscopic percutaneous internal ring suturing was generally achieved after approximately 30 procedures, after which operative performance tended to stabilize. To minimize the influence of learning-curve effects, all procedures in the present study were performed by the same experienced surgical team that had already completed the learning curve for advanced laparoscopic hernia repair techniques.

Although reinforcement-based procedures may offer potential advantages in selected patients with giant inguinal hernia, these techniques are technically more demanding and require advanced laparoscopic skills. During iliopubic tract suturing, particular attention should be paid to preserving the spermatic vessels and avoiding excessive narrowing of the internal ring in order to maintain adequate testicular perfusion. In theory, suturing in the vicinity of the iliopubic tract may also carry a risk of injury to the ilioinguinal or genitofemoral nerves, potentially contributing to chronic postoperative groin pain or sensory disturbance [24]. Accordingly, meticulous control of suture depth and avoidance of excessive tissue traction are essential during the procedure. Although no cases of chronic groin pain or sensory abnormality were identified during follow-up in the present study, these outcomes were not systematically assessed using validated long-term pain evaluation instruments.

Despite the increased technical complexity, no significant differences were observed among the three groups in the incidence of intraoperative subperitoneal hematoma, suggesting that the additional operative steps did not substantially increase bleeding risk when performed by experienced surgeons. All hematomas were minor, self-limited, and successfully managed with brief compression without compromising visualization of the spermatic cord structures.

Postoperative pain is another important consideration, particularly for procedures involving iliopubic tract suturing because of the tissue tension generated in this region. Some authors have suggested that additional suturing around the internal ring may theoretically increase postoperative discomfort [25]. However, our findings did not support this assumption. Postoperative pain was assessed using the FLACC scale, which has been validated for evaluating pain in infants and young children who are unable to reliably verbalize pain severity [8]. No significant differences in postoperative pain scores were observed among the three groups, and all patients experienced only mild postoperative discomfort that resolved spontaneously without additional analgesic intervention.

The most clinically relevant finding of this study was that no recurrence was observed in either reinforcement group during the 12-month follow-up period, whereas three recurrences occurred in the conventional single high ligation group (*P* = 0.015). Exploratory odds ratio analysis yielded findings consistent with the primary analysis. However, given the retrospective study design, potential treatment-selection bias, and the very small number of recurrence events, these findings should be interpreted cautiously and regarded as exploratory rather than confirmatory.

Several mechanisms may explain the observed difference in recurrence rates. Reinforcement of the internal ring through approximation of the iliopubic tract to the transversus abdominis arch may enhance closure stability by reducing the size of the internal ring and distributing local tissue tension more evenly. Similarly, double-loop high ligation may provide additional mechanical support through a second independent ligature. Nevertheless, the present study was not designed to directly evaluate the biomechanical mechanisms underlying recurrence prevention, and definitive causal relationships cannot be established.

All recurrent cases occurred within 6 months after surgery. The internal ring diameter in these patients was close to 2.0 cm, and one patient had a history of incarceration. Findings at reoperation suggested incomplete closure of the internal ring and increased local tension at the ligation site as possible contributing factors; however, these observations should be interpreted as descriptive findings rather than definitive explanations for recurrence.

In our institution, reinforcement-based procedures were preferentially selected when the internal ring diameter approached or exceeded 2.0 cm because larger defects were considered more likely to be associated with increased tension after ligation. However, the 2.0-cm threshold was derived primarily from institutional experience rather than formal cutoff optimization analysis. Therefore, this threshold should be regarded as exploratory and hypothesis-generating, and further validation in larger prospective studies is warranted before any definitive clinical recommendation can be made.

### Limitations

This study has several limitations. First, postoperative recurrence was uncommon, with only three recurrence events observed during follow-up. Although recurrence rates differed significantly among the three groups, the limited number of events reduced the precision of the estimated treatment effect. Although post hoc power analysis demonstrated acceptable statistical power, the limited number of recurrence events restricts the practical interpretability of this estimate. Accordingly, the recurrence findings should be regarded as exploratory rather than definitive. Second, this was a retrospective, non-randomized study, and treatment allocation partly depended on intraoperative assessment and surgeon judgment. Although an exploratory subgroup analysis was performed in patients with an internal ring diameter of 1.5–2.0 cm to partially reduce indication bias, residual confounding cannot be completely excluded. Because recurrence events were rare, multivariable regression analysis and propensity-score adjustment were not performed. Third, the 2.0-cm internal ring diameter used as an intraoperative decision-making threshold was based primarily on institutional experience and clinical observation rather than formal statistical cutoff optimization. Therefore, this threshold should be regarded as exploratory and hypothesis-generating rather than a validated criterion for clinical decision-making. Fourth, follow-up was limited to 12 months. Although all recurrences occurred within the first 6 months after surgery, late recurrence cannot be excluded. In addition, internal ring diameter measurement may have been subject to observer-dependent variability despite the use of a standardized measurement method. Postoperative pain was assessed only during the early postoperative period, and chronic postoperative groin pain, sensory disturbance, and other long-term patient-reported outcomes were not systematically evaluated. Despite these limitations, The present findings suggest that reinforcement-based laparoscopic procedures may be associated with a lower observed recurrence rate in selected children with giant inguinal hernia without increasing perioperative morbidity. Nevertheless, these findings should be interpreted cautiously. Larger prospective multicenter studies are warranted to validate these observations and to determine the optimal internal ring diameter threshold for surgical decision-making.

## Data Availability

The datasets generated and/or analyzed during the current study are available from the corresponding author on reasonable request.

## Abbreviations

LHL: Laparoscopic high ligation
SD: Standard deviation
IQR: Interquartile range
